# Explainable artificial intelligence identifies and localizes left ventricular scar in hypertrophic cardiomyopathy using 12-Lead electrocardiogram

**DOI:** 10.1038/s41598-025-09282-7

**Published:** 2025-09-30

**Authors:** Kasra Nezamabadi, Sanjay Sivalokanathan, Jiwon Lee, Talha Tanriverdi, Meiling Chen, Daiyin Lu, Jadyn Abraham, Neda Sardaripour, Pengyuan Li, Parvin Mousavi, M. Roselle Abraham

**Affiliations:** 1 Computational Biomedicine Lab, Computer and Information Sciences, University of Delaware, Newark, DE USA; 2 Division of Cardiology, Hypertrophic Cardiomyopathy Center of Excellence, University of California, San Francisco, 555 Mission Bay Blvd South, San Francisco, CA 94127 USA; 3 Hypertrophic Cardiomyopathy Canter of Excellence, Division of Cardiology, Johns Hopkins School of Medicine, Baltimore, MD USA; 4 Department of Biomedical Engineering, Vanderbilt University, Nashville, TN USA; 5 IBM Research – Almaden Lab, San Jose, CA USA; 6 School of Computing, Queen’s University, Kingston, ON Canada

**Keywords:** Left ventricular scar, Hypertrophic cardiomyopathy, Machine learning, Explainable AI, Unsupervised clustering, Self-supervised learning, Cardiology, Machine learning

## Abstract

Left ventricular (LV) scar is a major risk factor for sudden death and heart failure in hypertrophic cardiomyopathy (HCM). LV scar evolves over time and needs longitudinal assessment. Currently, LV scar detection relies on late gadolinium enhancement MRI, which is limited by high cost and artifacts from implanted cardiac devices. To address this, we developed *XplainScar*, an explainable machine learning model that identifies LV scar using 12-lead electrocardiogram (ECG) data. *XplainScar* was trained and validated on retrospective data from 748 HCM patients across two centers (500 from Johns Hopkins hospital for model development, and 248 from UCSF for validation). *XplainScar* employs a combination of unsupervised and self-supervised representation learning to effectively predict scar presence, and discover ECG features associated with LV scar. *XplainScar* rapidly analyzes ECG data (< 1 min for 10 patients) and demonstrates strong predictive performance on the held-out test set, achieving an F1-score of 89%, sensitivity of 90%, specificity of 78%, and precision of 88%. By providing an effective, cost-effective, and transparent alternative to MRI, *XplainScar* has the potential to assist with patient care, and reduce healthcare costs related to LV scar monitoring in HCM. *XplainScar* is available at https://github.com/KasraNezamabadi/XplainScar.

**Supplementary Information:**

The online version contains supplementary material available at 10.1038/s41598-025-09282-7.

## Introduction

Hypertrophic cardiomyopathy (HCM), the most common cardiac genetic disease and cause of sudden cardiac death in young individuals^1–5^, is characterized by variable penetrance and phenotypic heterogeneity^6–8^. The pathologic hallmarks of HCM are myocyte hypertrophy, myocyte disarray, cardiac fibrosis (scar), and microvascular remodeling. But the location/extent of left ventricular hypertrophy (LVH) and scar vary, even among individuals from the same family, and can evolve over time^9,10^. Longitudinal monitoring of LV scar and LVH by magnetic resonance imaging (MRI) is recommended by the AHA/ACC guidelines^9^ because high LV scar burden (> 15% of LV mass) and severe LVH (wall thickness > 3 cm) are risk factors for sudden cardiac death and heart failure^11–14^. However, the high cost and limited availability of MRI worldwide, as well as susceptibility to artifacts from implanted devices (e.g. pacemaker or defibrillator)^15^ complicate longitudinal scar monitoring by MRI. Hence there is need for MRI-independent methods for LV scar detection in HCM.

The 12-lead electrocardiogram (ECG) is an ideal candidate for LV scar detection, because it is widely available worldwide, relatively inexpensive, not influenced by defibrillator implantation, and reflects regional as well as global cardiac electrical activity, that can be impacted by the presence of LV scar. Specifically, the QRS complex reflects myocardial depolarization, whereas the ST segment and T wave reflect repolarization. LV scar would be expected to primarily impact impulse propagation in the LV, and hence the QRS complex. But, ischemic, dilated, and hypertrophic cardiomyopathies, which are associated with LV scar, can also have concomitant electrical remodeling^16^, leading to changes in the ST segment and T wave, in addition to QRS complex abnormalities. In the case of HCM, pathogenic variants in sarcomeric protein genes lead to myocyte hypertrophy, myocyte disarray, interstitial and/or replacement fibrosis, changes in ion channel/gap junction expression/function^17^ and/or coronary microvascular structure/function^18^, which can influence myocardial depolarization and repolarization^19^. This structural and/or electrical remodeling in HCM hearts leads to a variety of ECG abnormalities involving the QRS complex^20–23^, ST segment and T wave^21,24^ in several or all leads, that evolve over time. But unlike myocardial infarction where LV scar is typically transmural or endocardial, and in a vascular distribution, LV scar in HCM is often patchy, mid-myocardial and not in a vascular territory^25^. This could explain why Q waves which are highly predictive of LV scar in ischemic cardiomyopathy, do not predict LV scar in HCM^26^. Small studies in HCM patients suggest an association between QRS complex fragmentation^27^, low QRS voltage^28^, T wave inversion^26^ and strain pattern^27^ with LV scar. But specific methods to predict LV scar from 12-lead ECG data in an individual with HCM are lacking. We address this problem with a machine learning (ML)-based solution that learns from extensive HCM-ECG data to discern patient-specific ECG features indicative of LV scar. Our model *XplainScar*, extracts comprehensive features from routine 12-lead ECGs using an HCM ECG-specific segmentation algorithm^29^, and incorporates unsupervised as well as self-supervised representation learning, to yield an explainable ML framework that effectively predicts the presence or absence of LV scar, and reveals ECG markers associated with scar location, in < 1 min per 10 patient-ECGs.

We demonstrate that *XplainScar*, the first explainable machine learning method for HCM LV scar detection using ECG data, performs well and generalizes across different centers. *XplainScar* has the potential to reduce health care costs by using routine 12-lead ECG data for LV scar detection, and improve HCM patient outcomes by enabling longitudinal monitoring of LV scar, which is associated with greater severity of myopathy and adverse events.

## Methods

**Fig. 1:**
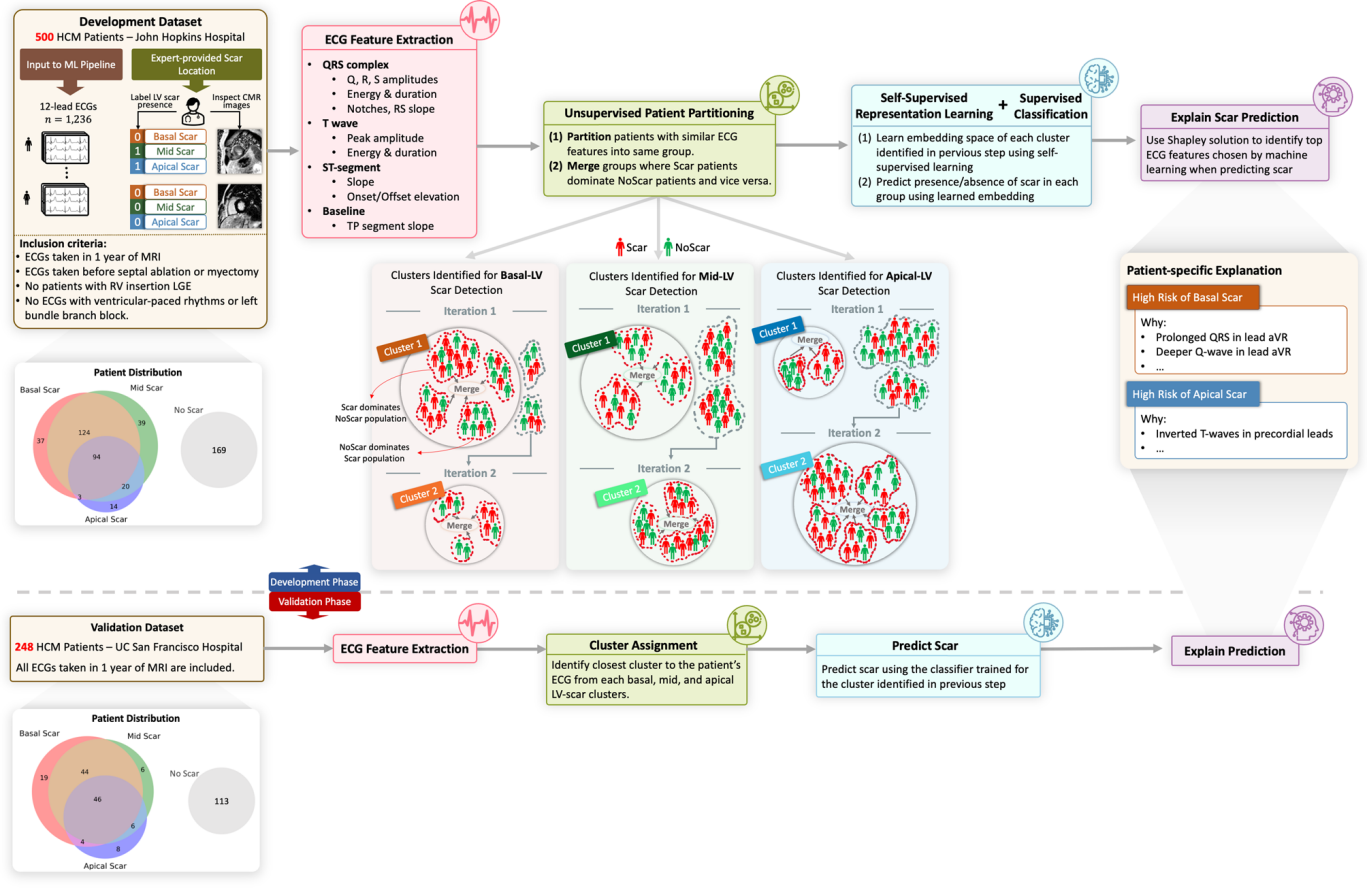
Overview of *XplainScar*. This method was developed using the JH-HCM dataset (*n* = 500), and validated using the UCSF-HCM dataset (*n* = 248), after excluding HCM patients with LGE at RV insertion sites. In each patient, the LV was divided into basal, mid, apical regions for scar (LV-LGE) detection. P, Q, R, S, T waves in 12-lead ECGs were identified using a segmentation method developedfor HCM ECGs. ECG features such as duration, amplitude, slope, energy of QRS complexe and T waves, as well as ST, TP segments were extracted from each lead, and adjusted for LV mass index, age, sex, using multiple linear regression. Subsequently, patients were partitioned into groups based on similarity of their ECG features using unsupervised clustering. In each group, a self-supervised neural network followed by a fully connected neural net predicted presence of LV scar. The Shapley value approach was used to identify the top ECG features that participated in LV scar prediction in the basal, mid and apical LV in each HCM patient.

The *XplainScar* framework (Fig. 1) employs a sequential pipeline analyzing ECG data to predict LV scar. To clarify its application, we first describe how it operates in a clinical setting for a new HCM patient, followed by details of its development and training process.

### Clinical application workflow

**Fig. 2:**
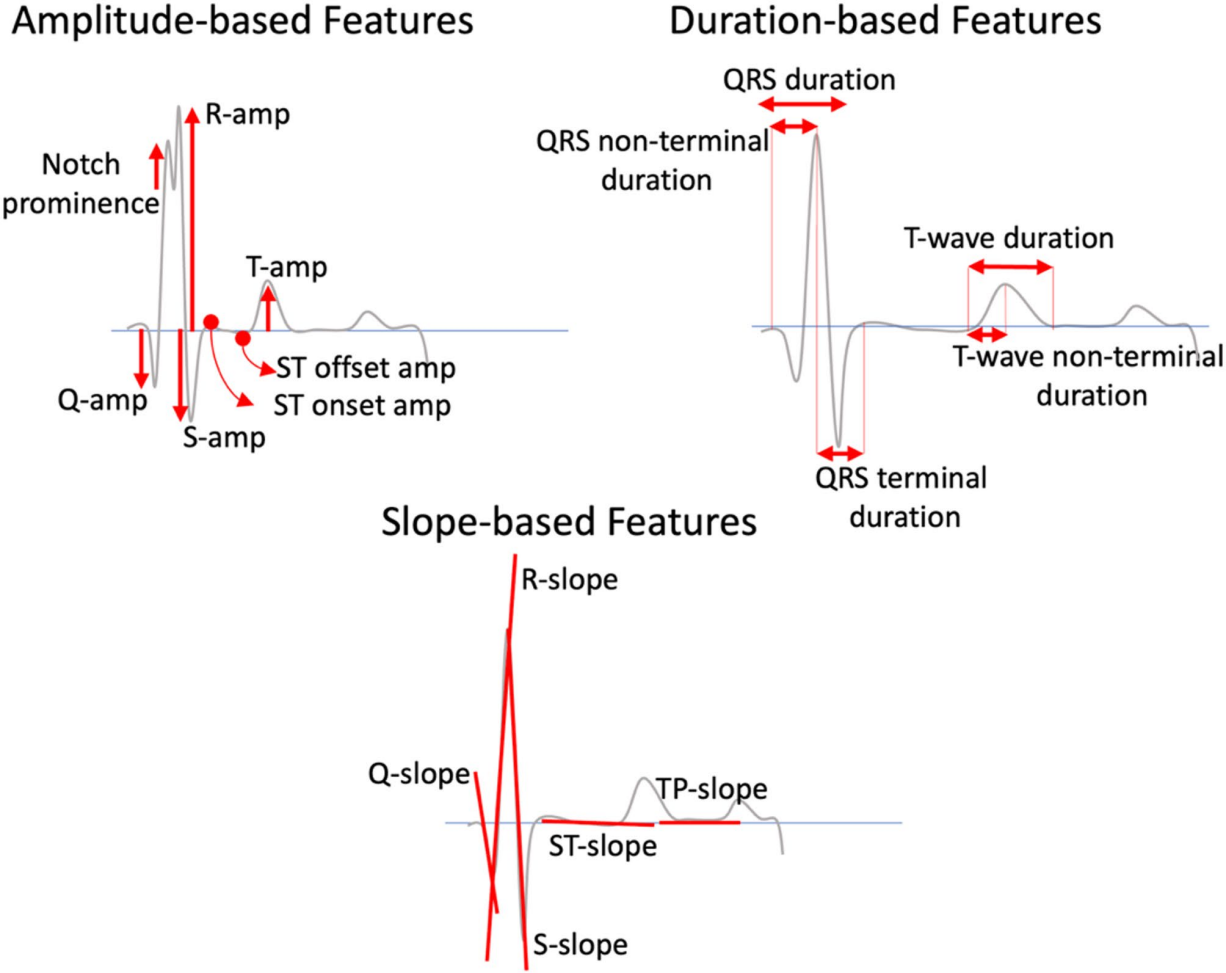
Features extracted from each lead of the input 12-lead ECG for LV scar detection by XplainScar. In addition to these features, the energy and area under the curve of QRS complex and T wave are also extracted from each lead.

In the clinical setting, *XplainScar* receives a standard 10-second 12-lead ECG from an HCM patient and outputs probabilities indicating scar presence in the basal, mid, and apical regions of the left ventricle (LV). The input ECG is first segmented to P, QRS, and T waves using a custom-built, and publicly- available segmentation method developed by our group,  for HCM-ECGs^29^. Next, a pre-defined set of ECG features representing ventricular depolarization and repolarization are automatically calculated from the segmented waves ( see *Step 1: Extract ECG features* section and Fig. 2 for details). The patient is then assigned to one of the pre-calculated patient clusters based solely on the similarity between their ECG features, and the cluster centroids learned during development. Each cluster has an associated neural network classifier, trained specifically for that group, which predicts the likelihood of scar presence in the basal, mid, and apical LV regions. To ensure transparency, the framework generates an explanation for the prediction using the Shapley value approach^30^. This method assigns an importance score to each of the input ECG features, indicating how strongly and in which direction (towards predicting *Scar* or *NoScar* ) each feature influenced the final output.

## Model development and training

### Patient population and data source

Cardiac magnetic resonance images (MRI) and 12-lead ECG from HCM patients at the Johns Hopkins ($\:n=500$) and University of California, San Francisco ($\:n=248$) HCM Centers of Excellence (HCM-COE) were used for model development and validation, respectively. All HCM patients underwent deep clinical phenotyping, consisting of rest/stress ECG and echocardiography (ECHO), cardiac MRI, rhythm monitor or device interrogation, labs, prior to their first clinic visit at the HCM-COE. MRI images were analyzed for late gadolinium enhancement (LGE) within 1 year of ECG to obtain the ground truth for LV scar; these labels were used for training *XplainScar.* Detailed demographic, clinical, ECG, and MRI characteristics, as well as our method for scar labeling, are provided in the supplemental materials (Supplemental Tables 1 and 2, Supplemental Fig. 1).

For training purposes, the presence of scar was treated as three independent binary labels, one for each of the basal, mid, and apical LV regions. This approach allowed for the inclusion of all patients, including those with scar spanning one, two, or all three regions, and enabled the model to learn ECG features specific to each location. For global LV scar determination, we applied an inclusive rule: any patient with scar in at least one of the three regions was labeled as LV scar-positive (OR logic across regional predictors, as outlined in Table 1).

**Table 1:** LV-scar detection performance by our method in terms of positive predictive value (PPV), sensitivity (Se), specificity (Sp), F1 score (F1), and 95% confidence interval around accuracy.

Region	John Hopkins Dataset # of patients = 500 (5-fold)					UCSF Dataset # of patients = 248 (left-out test)				
	PPV	Se	Sp	F1	95% CI Acc.	PPV	Se	Sp	F1	95% CI Acc.
Basal	94	87	90	90	86.2–87.4	88	85	82	87	81.3–82.7
Mid	91	87	85	89	86.4–88.3	85	85	83	85	86.1–87.5
Apical	89	85	91	87	85.6–86.9	88	82	92	85	84.6–85.7
Global LV Scar determination $\:basal\:\varvec{o}\varvec{r}\:mid\:\varvec{o}\varvec{r}\:apical$	90	95	80	92	86.9–88.0	88	91	78	89	84.5–86.1

$\:Sp=\frac{TN}{TN+FP}$, $\:Se=\frac{TP}{TP+FN}\:$, $\:PPV=\frac{TP}{TP+FP}$, $\:F1=\frac{TP}{TP\:+\:\frac{1}{2}(FP+FN)}$, where TP (True Positives) denotes LV scar correctly identified by *XplainScar*. TN (true negatives) denotes patients without LV scar on MRI who are not identified to have LV scar by *XplainScar*; FP (false positives) denotes patients who do not have LV scar by MRI but are misclassified by *XplainScar* to have LV scar. FN (false negatives) denotes patients who were incorrectly labeled by *XplainScar* to not have LV-scar.

Patients with ventricular-paced rhythms, and left bundle branch block (LBBB) were excluded from the JH cohort due to the impact of these conditions on ventricular depolarization/repolarization, and ECG morphology, which could confound model learning. However, in the left-out test set, the UCSF cohort, all ECGs recorded in the clinical setting—including those with ventricular-paced rhythms , incomplete blocks, and LBBB/RBBB—were included during evaluation. This allowed us to assess XplainScar’s generalizability to real-world ECG patterns, including those not represented during training. Furthermore, patients with poor quality MRI images, where LV-LGE could not be quantified reliably and confidently by an experienced cardiologist, and those with LGE confined *exclusively* to the right ventricular (RV) insertion points were excluded. This latter exclusion was based on evidence suggesting RV insertion point LGE may not represent myocardial scar.

### Step 1: extract ECG features

For each heartbeat segmented across the input 12-lead ECG, we extract 23 features representing ventricular depolarization and repolarization from the QRS complex, ST segment, T wave, TP segment (Fig. 2). Additionally, we compute the energy and area under the curve (AUC) of QRS complex and T wave. We deliberately chose these basic, interpretable features over more complex, predefined ECG criteria (like QRS scores) to mitigate potential bias, and allow the model to learn data-driven feature combinations relevant to LV scar (Supplemental Table 3).

Each lead is then represented by a 23-dimensional feature vector $\:V\:=\:<{\stackrel{-}{v}}_{1},\:{\stackrel{-}{v}}_{2},\:\dots\:,{\stackrel{-}{v}}_{23}>$ where $\:{\stackrel{-}{v}}_{i}$ is the average of the feature $\:{v}_{i}$ in all heartbeats in the lead. By concatenating the feature vectors of the individual leads, a 12-lead ECG is represented by a $\:12\:\times\:\:23=276$-dimension vector. As such, each patient $\:{p}^{i}$ where $\:1\:\le\:i\:\le\:500$ in the JH dataset, and $\:1\:\le\:i\:\le\:248$ in the UCSF dataset, is represented as $\:m\:\times\:\:276$ feature matrix, where $\:m$ denotes the number of ECGs recorded from the patient within one year of MRI ($\:m=3\:\pm\:2$ in the JH dataset, and $\:m=2\:\pm\:1$ in the UCSF dataset). This results in a $\:1236\:\times\:\:276$-dimension JH dataset, and a $\:890\:\times\:\:276$-dimension UCSF dataset.

### Step 2: adjust for confounders (LV mass index, age, sex)

We observed a correlation between LV mass index ($\:LV\:mass/body\:surface\:area$) and LV scar percentage (Pearson correlation *r* = 0.4, *p* < 0.01) and QRS energy in lead V4 (Pearson correlation *r* = 0.6, *p* < 0.01). Since confounder bias can cause inaccurate estimations of associations due to its influence on both dependent and independent variables, we adjusted our dataset for age, sex and LV mass index (LVMI) by multiple linear regression. For each ECG feature, a linear regression model trained on the JH dataset estimates the feature value, using the patient’s LVMI, age, sex. Note that this adjustment is meaningful only during the training phase to avoid introducing biases into the model. During the testing and deployment phases predictions rely exclusively on the raw ECG feature values.

### Step 3: partition patients based on ECG features, using unsupervised clustering

We observed that no machine learning method trained on the JH dataset, using either raw ECG signals or features computed in previous steps, could effectively separate patients with LV scar (Scar group) from patients without LV scar (NoScar group) (F1 score < 0.7, Table 2). To address this, we developed a method to partition patients into more homogeneous subgroups based on ECG similarity, facilitating more effective learning within each group. We recursively (1) partition patients with similar ECG features into the same group (unsupervised clustering) and (2) merge groups where the Scar group dominates the NoScar group, and vice versa, until all patients are assigned to a merged group (Fig. 1).

**Table 2:** LV scar detection performance obtained by our method compared to five state-of-the-art methods and two ablation experiments over the JH dataset in terms of F1 score.

Method	F1 score	Overview
Melgarejo^57^	69	
Dima^58^	58	
Goto^34^	57	
Ko^35^	51	
Che^36^	63	
*XplainScar* No Self-supervised	84	
*XplainScar* No Partitioning	41	
*XplainScar* Full pipeline	**92**	

SVM: Support Vector Machine; FCN: Fully Connected Neural Network; 2D CNN: 2-Dimensional Convolutional Neural Network.

To partition patients, we used the Dirichlet Process Gaussian mixture model, a distribution-based clustering algorithm that automatically learns the number of clusters^31^. After partitioning the patients into separate groups, we merge groups where the Scar group dominates the NoScar group, and vice versa. We initially consider a class dominating another if its population is at least twice the other class’s population within the cluster. During next recursive iterations, we reduce the domination ratio by 50% to favor less identified groups over more separation. Notably, our proposed recursive clustering algorithm only iterated twice over the JH dataset, resulting in two merged groups of patients for each of the basal, mid, and apical LV scar detection tasks (Supplemental Algorithm 1).

### Step 4: predict Scar using self-supervised and supervised machine learning

For each resulting cluster, we train a self-supervised neural network called SCARF (Self-supervised Contrastive Learning using Random Feature Corruption)^32^ to predict the presence of scar in the basal, mid, and apical LV. SCARF effectively learns underlying patterns from data without relying heavily on labeled samples, which is particularly beneficial given the complexity and variability of HCM-ECG patterns^29,33^. SCARF training has two steps: *(1)* self-supervised representation learning, and *(2)* supervised binary prediction.

In the first step, a task-agnostic representation of the input ECG feature space is learned without expert-provided labels. For each ECG $\:{x}^{\left(i\right)}$ from the JH dataset, a *corrupted view* $\:{\stackrel{\sim}{x}}^{\left(i\right)}$ is generated by sampling 60% of $\:{x}^{\left(i\right)}$ features uniformly at random and replacing them with a random draw from the uniform distribution, over the values that features take on across the training. An encoder $\:f$ is then trained on a contrastive loss to generate a lower dimension embedding space $\:Z$. This loss function forces $\:f$ to generate pair ($\:{z}^{\left(i\right)}$, $\:{\stackrel{\sim}{z}}^{\left(j\right)}$) that are close if $\:i=j$ and far apart if $\:i\ne\:j$.

Next, a fully connected neural network $\:h$ is attached to the learned encoder $\:f$ to predict the presence of LV scar using the embedding space $\:Z$. Both $\:h$ and $\:f$ are tuned to minimize the cross-entropy classification loss following 5-fold cross validation over the JH dataset. Given an unseen test ECG feature vector $\:{x}_{test}^{i}$, the encoder $\:f$ first maps $\:{x}_{test}^{i}$ to an embedding space $\:{z}_{test}^{i}$ and then the classifier $\:h$ decides whether $\:{z}_{test}^{i}$ represents scar.

### Step 5: explain model predictions using Shapley additive explanation (SHAP)

**Fig. 3:**
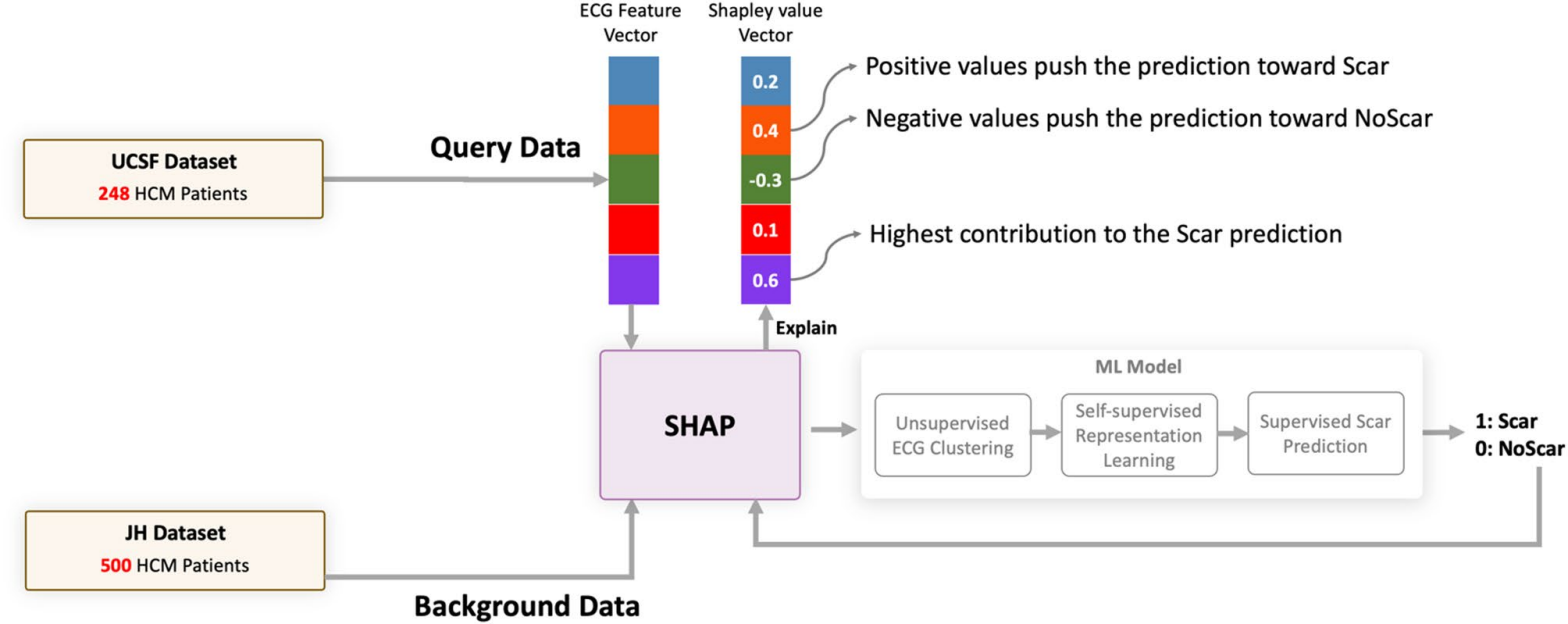
Overview of the explanation framework in *XplainScar*. We feed the entire JH dataset as the training set to Kernel Explainer and use the Shapley values for each ECG from the UCSF dataset for LV scar prediction.

To ensure model transparency, we integrated the Shapley Additive Explanation (SHAP) framework to provide insights into why the model makes a specific prediction for an individual patient (Fig. 3). For any given prediction, SHAP calculates an importance score (Shapley value) for each of the 276 input ECG features. This score quantifies the contribution of that specific feature to the model’s output (pushing towards Scar or NoScar) for the patient. This allows clinicians to understand the factors driving individual predictions. Technical details regarding the SHAP implementation and analysis are provided in the Supplementary Materials.

### Experimental setup

Model development and hyperparameter tuning were conducted on the JH dataset using a 10-run, 5-fold cross-validation scheme. This involves repeatedly splitting the JH dataset into training and internal validation sets to ensure the model’s performance estimates are stable and not overly dependent on a single data split. The UCSF dataset was strictly held out during development and served as a completely independent, external test set for final validation of the model’s generalizability. We evaluate model performance by computing specificity, sensitivity, positive predictive value, F1-score and 95% confidence interval for accuracy.

## Results

### ***XplainScar*** can effectively detect LV scar using 12-lead ECG

Model performance for regional (base, mid, apex) and global LV scar detection is presented in Table 1. XplainScar achieved an F1 score of 0.92, sensitivity of 0.95, specificity of 0.8 over the JH dataset, and an F1 score of 0.89, sensitivity of 0.91, specificity of 0.78 over the left-out UCSF dataset. These results demonstrate the effectiveness of *XplainScar* in accurately identifying LV scar while highlighting its generalizability to unseen UCSF data. The small decrease in model performance when transitioning from the JH dataset to the UCSF set could result from factors such as differences in data collection methods (MRI scanners – 1.5T Siemens at JH, 3T GE at UCSF) between the two centers. Notably, *XplainScar* requires < 1 min (wall clock) to predict LV scar in 10 HCM patient ECGs, using one 10-second 12-lead ECG per patient, on a personal computer (8 GB of RAM memory with no GPU). This swift processing time and robust performance on unseen UCSF data underscore the potential for practical deployment of *XplainScar* in real-world scenarios to assist with disease management in the clinical setting.

### Ablation experiments and comparison with state-of-the-art methods

We performed ablation experiments (Table 2) to examine the importance of the unsupervised ECG clustering and self-supervised neural network steps in determining model performance (F1 score). Elimination of the patient clustering step led to ~ 50% reduction of the F1 score, and elimination of self-supervised representation decreased the F1 score by ~ 10%. These results indicate that patient partitioning is a key step in our pipeline for LV scar detection.

We did not find any deep learning approaches for ECG-based LV scar detection in HCM, so we leveraged state-of-the-art ECG-based deep neural networks proposed for HCM diagnosis^34,35^ and general arrhythmia detection^36^ for comparison (Table 2). The datasets used in these studies and the trained weights of the models are not publicly available; as such, we train the models over the JH dataset and test them following the same cross-validation scheme used by our method. To compensate for the relatively small size of the JH dataset, which can limit training of deep learning models, we synthetically generate 12-lead ECGs by employing the data augmentation method proposed by Gopal et al.^37^, where the 12-lead ECG is first transferred into 3D vectorcardiogram space using inverse Dowers transformation^38^, randomly rotated, and then converted back to the ECG space. Furthermore, as the exact architecture of the compared deep models may not be optimal for LV scar detection (since they were originally proposed for disease diagnosis), we perform an extensive grid search to obtain the best hyperparameters, including the size and number of convolution kernels, attention heads, and layers. All the methods’ preprocessing steps, including ECG denoising and re-sampling, and feature extraction (for non-deep-learning methods) are implemented as the authors propose. Notably, *XplainScar* significantly outperforms all 5 methods (Table 2).

### XplainScar reveals ECG features of regional LV Scar in HCM

Each prediction is explained as a series of ECG features and Shapley value pairs where higher values show that the corresponding feature significantly impacts the prediction (Fig. 3). We utilize the entire JH dataset as the training set for SHAP, which enables us to compute Shapley values for each prediction made on the UCSF dataset. Table 3 shows the average Shapley value of each ECG component and corresponding ECG leads across all predictions. *XplainScar* identified several QRS-related features in leads I, V1, aVR as predictors of basal scar, and T wave related features in leads V4 – V6, for mid and apical LV scar detection.

**Table 3:**
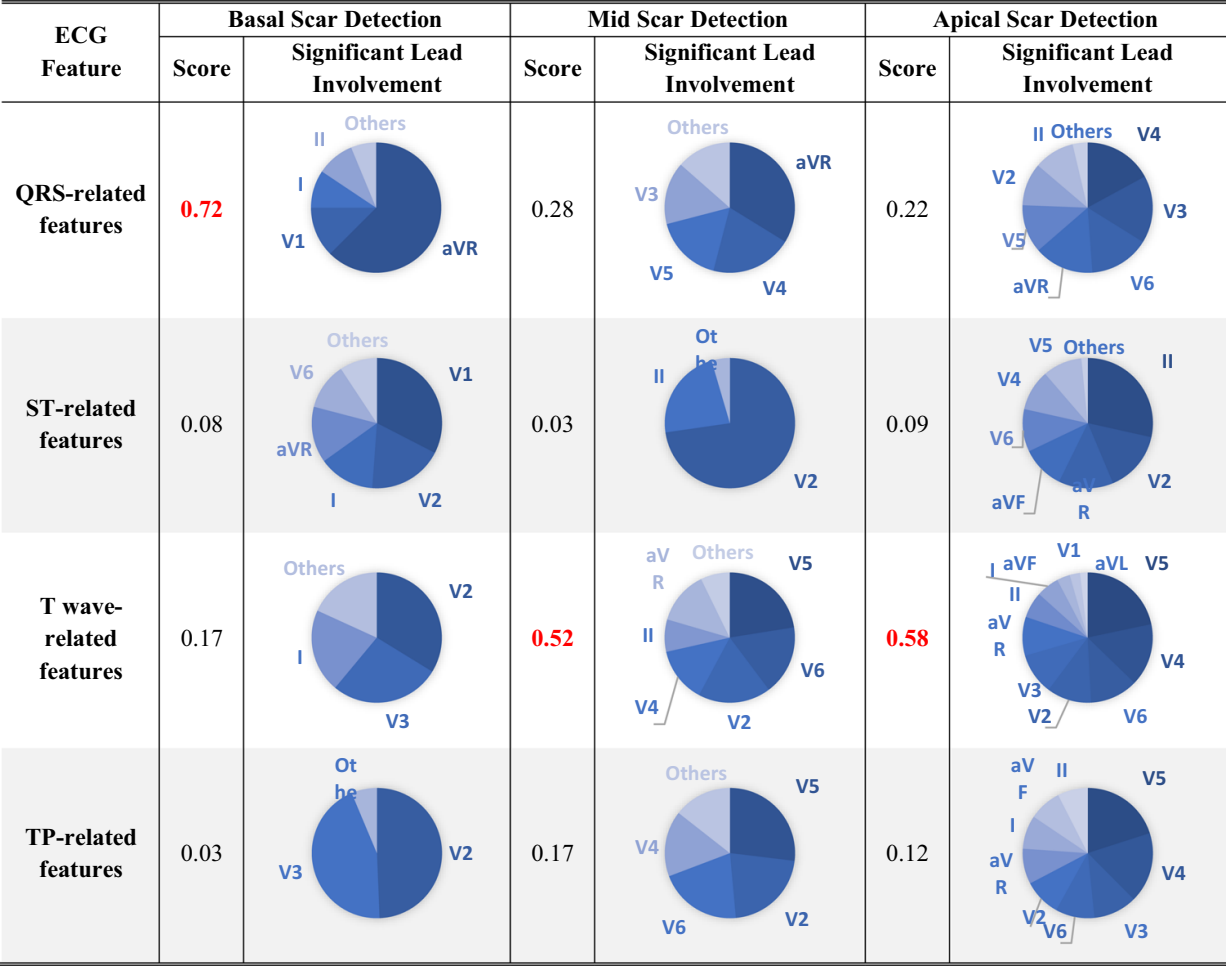
ECG feature importance score calculated as the average Shapley value of each ECG component across all predictions in the UCSF dataset along with significant leads involvements.

To reduce the sensitivity of our analysis to large variations in Shapley values generated by SHAP for regional scar prediction, we count the number of predictions where the specific ECG feature appeared within the top 20% of Shapley values, and report those with the highest frequencies (instead of averaging Shapley values of a feature across predictions) (Table 4). The mean and standard deviation for each feature in the Scar group compared with the NoScar group, using Welch’s T-test, are presented in Table 4. The scatter plot (Table 4) further illustrates how the value of each feature impacts the model output, pushing it toward predicting Scar or NoScar. For example, the first row of Table 4 shows that a deeper Q wave pushes *XplainScar* toward predicting basal scar, whereas absence of Q is associated with absence of basal scar (each dot in scatter plots corresponds to a single prediction).

*XplainScar* utilizes several ECG features for a single prediction, with only the most frequently used ones presented in Table 4. Top ECG features frequently selected for basal LV scar include deeper Q waves, prolonged non-terminal QRS duration, less negative area under the QRS complex curve (i.e. smaller area below the x-axis reflecting less negative amplitude) and smaller T wave amplitude in leads V1-V2. For apical LV scar, features such as T wave inversion, positive QRS complex area, and slope variations of the TP segment are prominent.

**Table 4:**
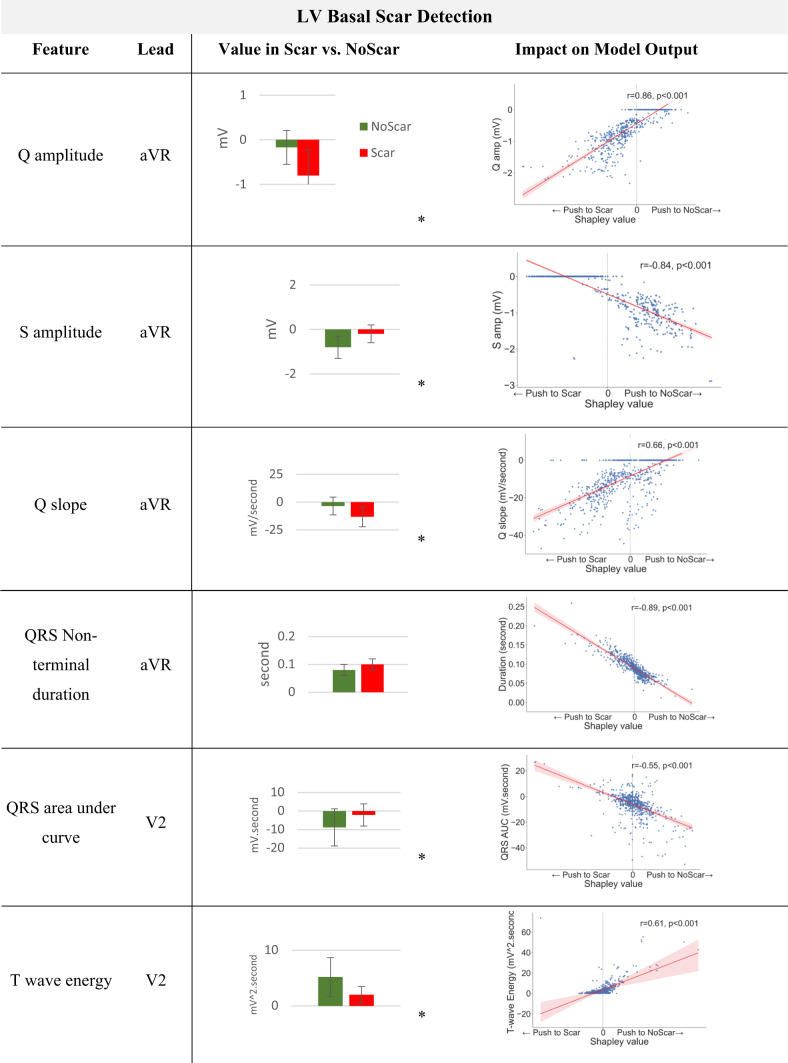

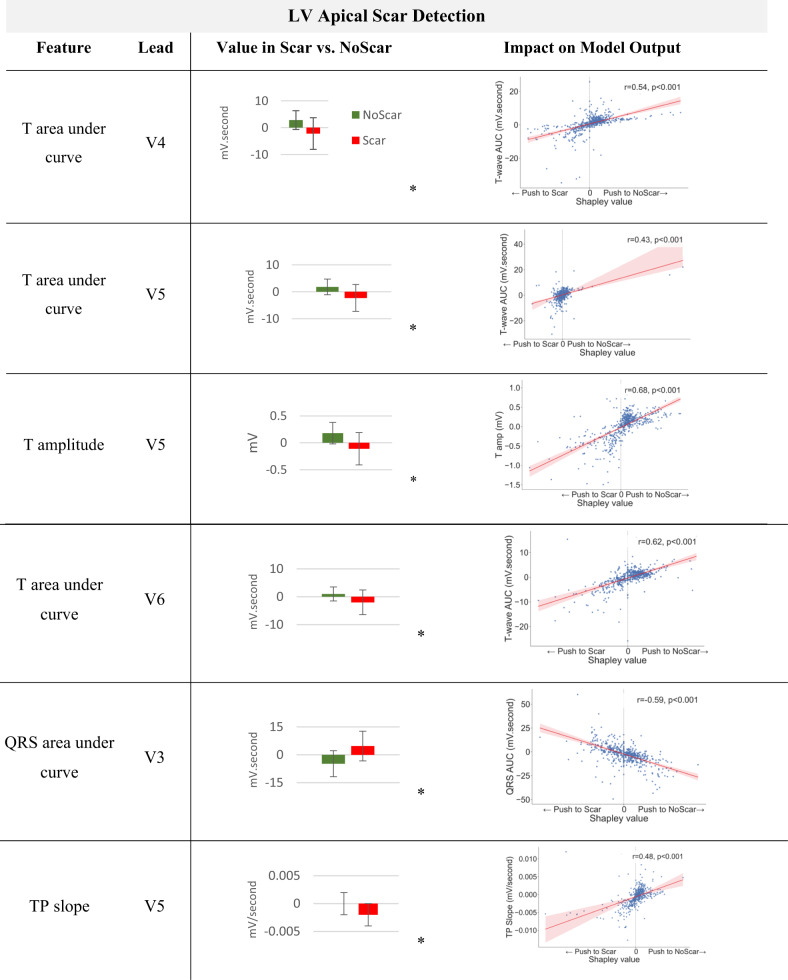
Top ECG features frequently selected by *XplainScar* when detecting basal and apical LV scar. Asterisk (*) shows statistically significant difference (*p* < 0.05) between feature values in Scar vs. NoScar groups, obtained using Welch’s T-test. Each Dot in scatter plots corresponds to a single prediction by *XplainScar*. These plots show how the value of a feature impacts *XplainScar* output.

### LV scar burden influences *XplainScar* performance and selected features

**Fig. 4:**
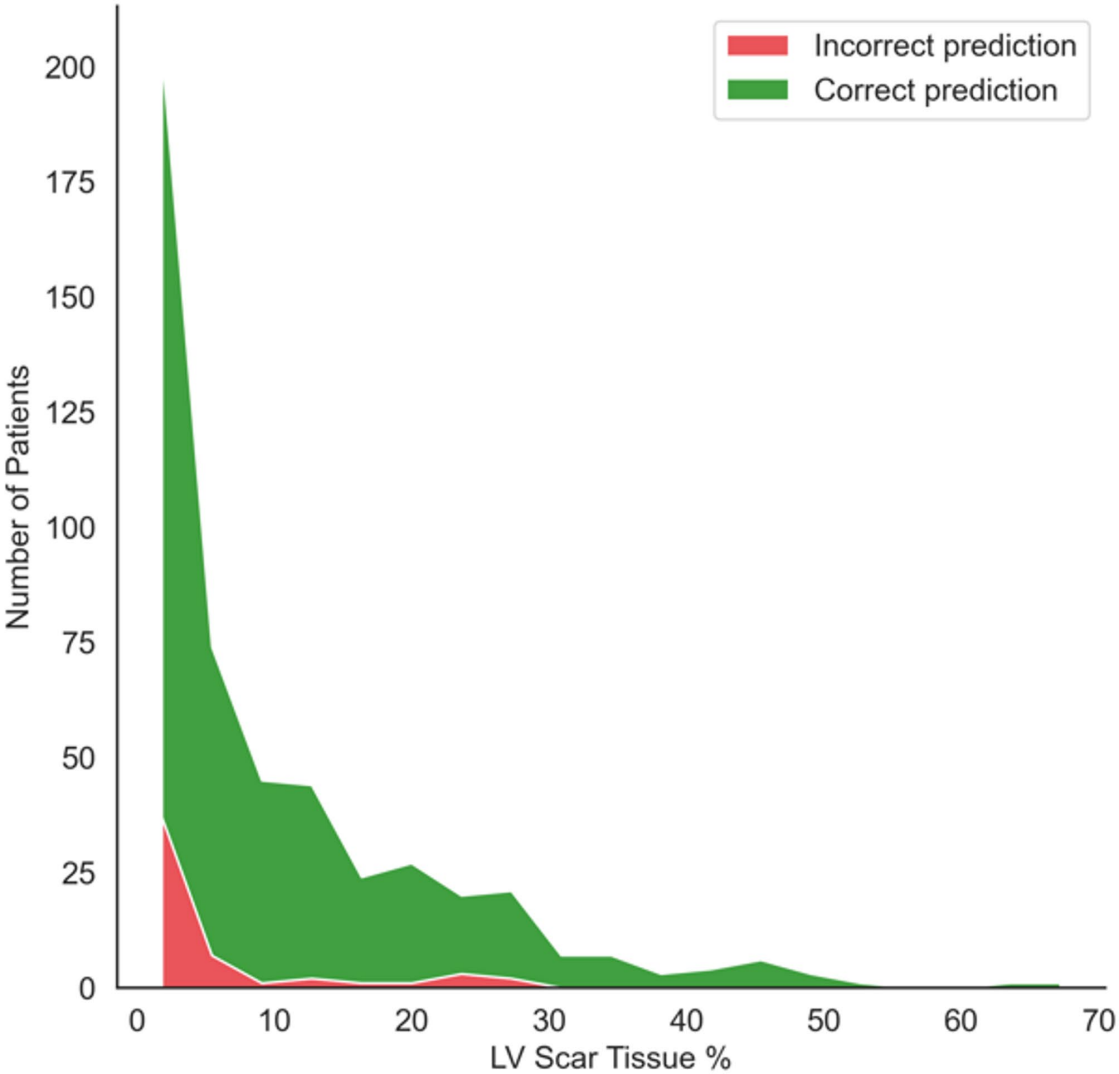
Performance of *XplainScar* over the JH dataset stratified by LV scar burden (scar mass as percentage of LV mass) quantified by MRI.

We perform a stratified analysis to test our method’s performance based on the percentage of LV scar mass, relative to total LV mass (Fig. 4). *XplainScar* achieves near-perfect detection in patients with scar percentage exceeding 10%. However, as scar burden decreases, there is a proportional increase in missed detections.

LV scar burden also influences the selection of ECG feature by *XplainScar.* Table 5 compares frequently selected ECG features by *XplainScar* with LV scar < 15% as well as > 15% of LV mass, and Table 6 compares the value of these features between the two groups. We chose the threshold of 15% based on previous findings that demonstrated increase in sudden cardiac death risk with LV scar > 15% of LV mass^39^. As scar burden increases, we observe a higher frequency of T wave inversion across multiple leads; Q amplitude was selected 14% more frequently in patients with LV scar burden > 15%, and QRS non-terminal duration was chosen 10% of the time in patients with LV scar burden < 15%.

**Table 5:** ECG features frequently selected by *XplainScar* stratified by LV Scar percentage.

Feature	LV scar ≥ 15% of LV mass		LV scar < 15% of LV mass	
	Appear among top 10 features?	Frequency of appearance	Appear among top 10 features?	Frequency of appearance
Q amplitude	Yes	37%	Yes	23%
QRS terminal duration	Yes	23%	Yes	8%
QRS non-terminal duration	No	–	Yes	10%
QRS area under curve	Yes	35%	Yes	17%
QRS energy	Yes	16%	No	–
T-wave inversion in chest leads	Yes	53%	Yes	28%
T-wave inversion in limb leads	Yes	38%	Yes	15%

**Table 6:** Comparison of ECG feature values from selected leads, stratified by LV Scar percentage.

Feature	Lead	Unit	Value in patients with ≥ 15% LV scar	Value in patients with < 15% LV scar	*p* value
Q amplitude	aVR	mV	− 0.6 ± 0.5	− 0.43 ± 0.51	0.002
QRS terminal duration	aVR	second	0.039 ± 0.022	0.038 ± 0.019	0.85
QRS non-terminal duration	aVR	second	0.091 ± 0.024	0.091 ± 0.024	0.95
QRS area under curve	V4	$\:mV.second$	1.8 ± 9.7	1.1 ± 8.6	0.2
QRS energy	V5	$\:{mV}^{2}.second$	18.6 ± 20.3	14.4 ± 16.5	0.001

### Case studies

**Fig. 5:**
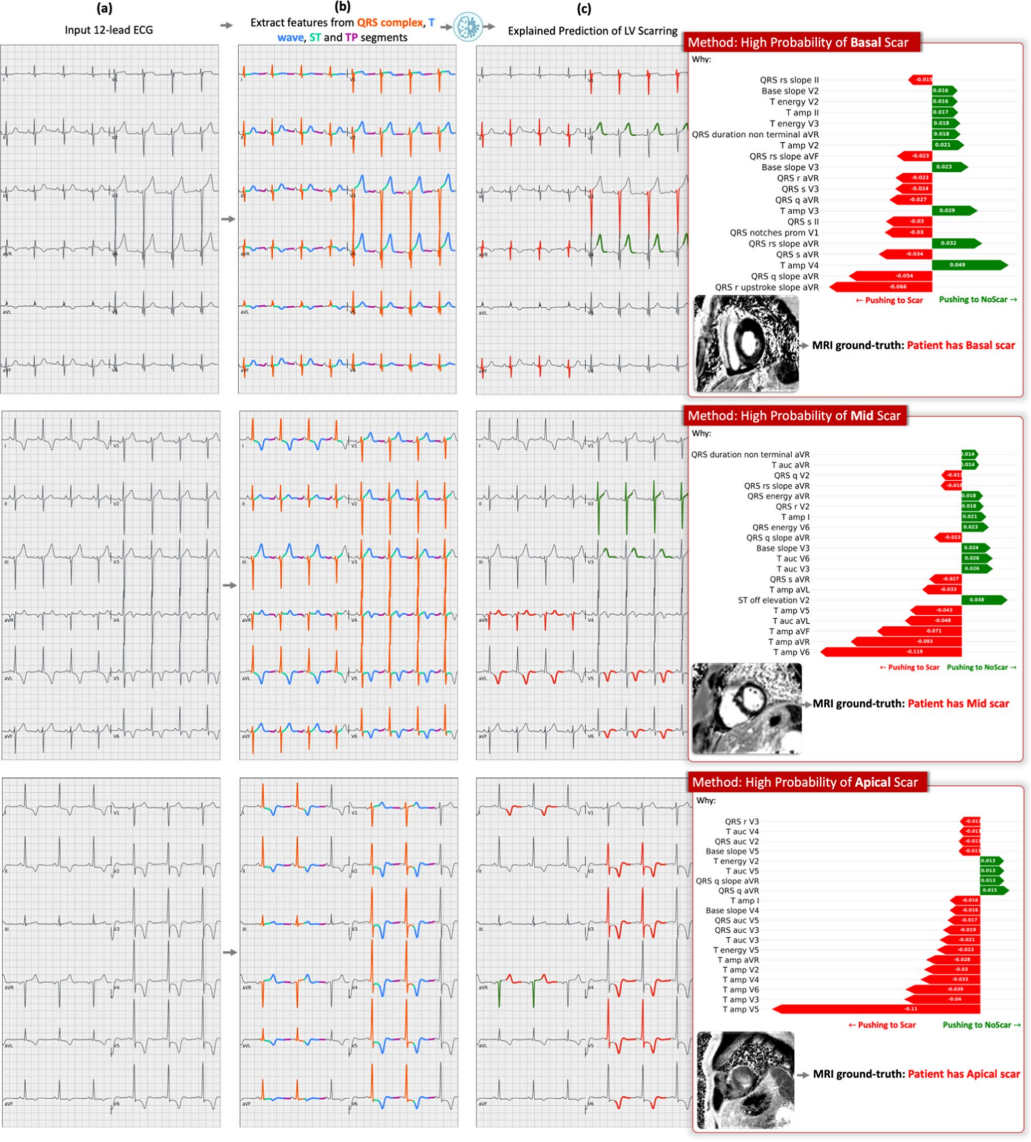
Case studies. Explained prediction of LV scar in 3 representative ECGs from the UCSF dataset by XplainScar. (**a**) Input 12-lead ECG, (**b**) Segmented ECG: QRS complex (in orange), ST segment (in green), T-wave (in blue), TP segment (in purple), (**c**) Our method’s prediction along with the ground truth label and the top 20% Shapley values showing the importance of each ECG feature in the prediction. The ECG component pushing the method towards predicting scar is highlighted in red on the 12-lead ECG, whereas those pushing the method toward NoScar are highlighted in green.

*XplainScar* is tailored to explain a single prediction. That is, given an ECG, *XplainScar* predicts the presence of scar in basal, mid, and apical regions of the LV, and explains why such a prediction is made (Fig. 5). Column-b of Fig. 5 illustrates the automatic annotation, and segmentation of the QRS complex, ST segment, T wave, TP segment, whereas column-c summarizes the ECG features with the top 20% of Shapley values, that drive scar prediction. In ECG #1, *XplainScar* used R upstroke slope in aVR, absence of S wave ($\:{S}_{amplitude}=0\:mV$), deep S wave in lead V3, fragmentation of QRS complex in V1 to predict basal-LV scar. In ECG #2 and ECG #3, scar prediction in the mid and apical LV, respectively, was driven by T wave features. T wave inversion in V2 – V6 was prominent in apical scar prediction, and T wave amplitude in V5, V6, aVL, aVR for scar detection in mid-LV.

### Correlation with EHR, ECHO, MRI data

**Fig. 6:**
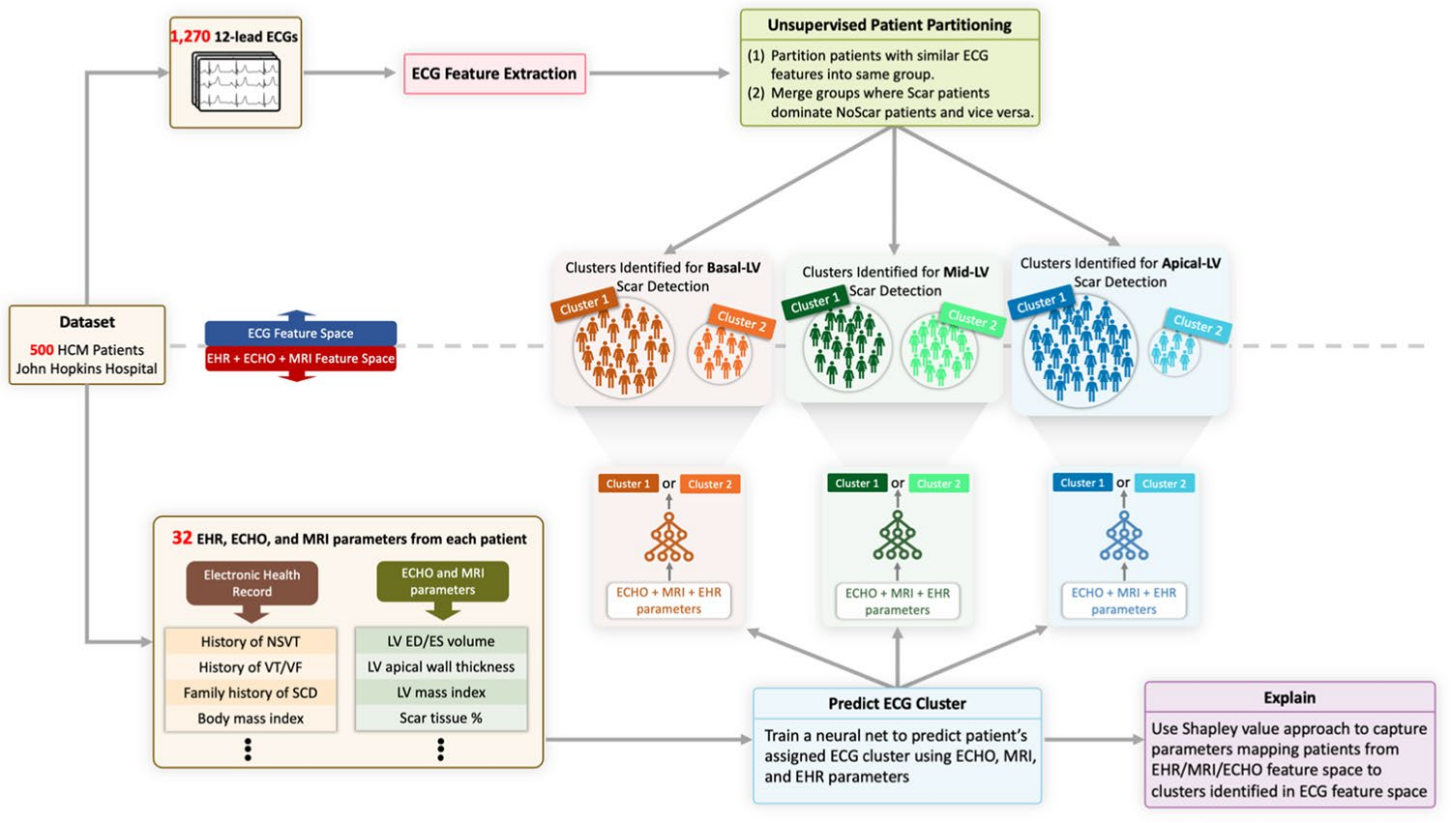
Clusters of HCM patients identified using ECG clustering correlate with EHR, ECHO, MRI data. Supervised neural networks trained in EHR, ECHO, and MRI feature space can effectively separate HCM patients into the same groups obtained by unsupervised patient partitioning step using ECG data.

We explored associations between patient clusters identified using ECG features and clinical/imaging features. To achieve this, we trained fully connected neural networks using 32 clinical variables (Supplemental Table 2) obtained from electronic health records (EHR), echocardiography (ECHO), and MRI data available for all patients in the JH dataset. These features are inputted into three fully connected neural networks trained on the JH dataset, learning a binary classification to identify patient cluster labels assigned during ECG partitioning (Fig. 6) for each basal, mid, and apical LV scar detection task. Each network performs binary classification, mapping EHR, ECHO, MRI parameters to cluster labels, evaluated using a 10-run 5-fold cross-validation scheme, with two clusters identified for basal, mid, and apical LV scar detection tasks.

Following a 10-run 5-fold cross-validation scheme, we observe that the neural networks perform well, with an average F1 score of 0.86 ± 0.03. This result suggests that patient clusters, initially identified solely through ECG analysis, can also be effectively separated using EHR, ECHO and MRI parameters, with the trained neural networks providing insights into this separation. The results presented in Table 7 highlight the top 5 clinical parameters selected by the neural networks to predict the cluster labels. The Shapley value of each parameter, and their value (mean and standard deviation) in each cluster are shown. Notably, univariate analysis does not reveal any statistically significant differences in the values of clinical parameters between the clusters. However, the neural networks successfully capture the correlations among several clinical parameters, enabling the mapping of patients from the EHR, ECHO and MRI feature space to their respective cluster labels found in the ECG feature space.

**Table 7:** Top 5 clinical parameters selected by supervised neural networks to predict the cluster labels for basal, mid, and apical Scar detection tasks using EHR, ECHO, MRI data, presented in descending order of Shapley values.

Predict the two clusters found for Basal LV scar detection		Predict the two clusters found for Mid-LV scar detection		Predict the two clusters found for Apical-LV scar detection	
Parameter	*P* value	Parameter	*P* value	Parameter	*P* value
Scar tissue %	0.1	History of obstructive CAD	0.4	Scar tissue %	0.05
LV end-systolic volume	0.7	Maximal interventricular septum thickness	0.06	LV apical wall thickness	0.7
History of NSVT	0.9	LV diastolic function	0.5	Mitral valve early-stage flow velocity	0.9
Maximal interventricular septum thickness > 3 cm	0.2	History of NSVT	0.2	LV mass	0.02
History of diabetes	0.25	Early mitral inflow deceleration time	0.09	History of smoking	0.2

These clusters are identical to those identified in the unsupervised patient partitioning step using solely patients’ ECG features. *P* values are calculated using welch’s t-test measuring the significance of difference for each parameters value between the clusters.

### Longitudinal testing

Since LGE represents cardiac replacement fibrosis, which is irreversible, we hypothesized that *XplainScar* would continue to detect LV scar in the same location, using ECGs obtained several years after initial ECG/MRI. To test this hypothesis, we identified 124 patients in the JH dataset who had ECGs recorded ≥ 4 years after MRI, with $\:86/124$ having LV scar based on MRI. *XplainScar* detected LV scar in 74/86 patients with LV scar detected by MRI, and $\:13/35$ patients who had no evidence of LV scar by MRI, at the time of their first clinic visit. However, none of these 13 patients had follow up MRIs, so we are unable to confirm that they developed LV scar over their follow up period. Prospective testing of our method with MRI is needed to demonstrate the utility of *XplainScar* for longitudinal monitoring of LV scar in HCM.

## Discussion

We developed a machine learning framework, *XplainScar* that is capable of handling diverse ECG patterns within a large HCM patient cohort, for effective LV scar detection and explanation. Our approach utilizes two innovative strategies to handle the significant heterogeneity of HCM-ECG patterns.

The first strategy is to extract basic yet comprehensive ECG features rather than *compound* ones such as the Selvester QRS score^40^ and fragmented QRS complex. Such compound features are calculated based on a set of basic, simple ECG criteria, but this calculation is often challenging even for human experts, and precludes discovery of new ECG features of LV scar. For instance, Vandenberk et al.^41^ reported significant interobserver variability (Kappa of 0.65) among five experienced observers when identifying fragmented QRS in 100 ECGs. Compound ECG features can also introduce bias to the input feature space, causing machine learning to learn less from other features that might be informative but not captured by the compound features. *XplainScar* uses a wide range of basic ECG features, based on physiological, biophysical and mathematical principles, to predict LV scar. By avoiding compound features and utilizing easily interpretable metrics, such as wave energy and amplitude, our method captures important markers for LV scar detection. Notably, our analysis agrees with previous studies regarding the significance of T wave inversion in apical LV scar^42,43^. Our method also uncovers previously unexplored ECG markers of LV scar, such as T wave energy, area under QRS, and Q amplitude, which play a significant role in detecting basal scar.

Our second strategy addresses HCM-ECG heterogeneity through a combination of unsupervised clustering and self-supervised representation learning. Unsupervised ECG clustering has been successfully employed to overcome challenges facing supervised ECG learning classification;  for instance, to resolve the imbalanced data problem, and low-level automation of patient-specific ECG classifiers^44^. Motivated by these applications, we propose a novel yet simple unsupervised approach to partition patients into more homogeneous HCM patient groups based on ECG similarity, improving the separation between those with and without LV scar.

Although we identify groups of HCM patients with high separation between the Scar and NoScar classes, an informative ECG representation discriminating the two classes is still needed. Such representation is challenging to learn, as the number of training samples and scar labels to train a performant classifier is limited in each group. Our self-supervised representation learning addresses this challenge. Self-supervised learning has proved effective in classifying raw ECG signals into abnormality classes despite scarce expert-provided labels^45–47^. Here, we employ it to learn a useful representation from tabular ECG features in each group, facilitating downstream scar classification.

Our ablation experiments clearly demonstrate that the combination of unsupervised ECG clustering and self-supervised representation learning is crucial for effective LV scar detection. Importantly, the groups of patients identified by unsupervised ECG clustering are not arbitrary partitions, but exhibit shared clinical parameters, even though these parameters were not seen during clustering. Notably, a supervised neural network trained on patients’ EHR, ECHO and MRI data successfully segregates HCM patients into the same groups identified by ECG clustering. This mapping of patients from one modality to another provides an extra layer of validation for *XplainScar*. The incorporation of clinical parameters and the correlations captured by the neural networks can pave the way for enhanced LV scar detection and personalized HCM patient management. By integrating information from multiple modalities, *XplainScar* facilitates a deeper understanding of the complex relationships between ECG patterns, clinical parameters, and LV scar in HCM. The combination of unsupervised ECG clustering and self-supervised representation learning, and the bridging between multiple modalities are novel approaches that have not been investigated before in other applications of computational ECG analysis. Thus, our work opens new avenues for future research in the HCM field.

A key strength of *XplainScar* lies in its explanation framework, which provides ECG signatures of basal, mid and apical LV scar in HCM, and thus prompts user trust in our method. Our explanation framework also sheds light on the inherent combinatorial non-linear power of *XplainScar*. Through this framework, we have discovered important ECG features of LV scar that do not exhibit a significant difference between the Scar and NoScar groups but are frequently selected by *XplainScar*. For example, non-terminal QRS duration (also called intrinsicoid deflection^48^) is frequently selected by our method when predicting basal-LV scar, but does not exhibit a significant difference between Scar and NoScar groups ($\:p=0.12$). We use SHAP to explain our model’s predictions of LV scar; SHAP employs a linear function that assigns importance scores to individual features but does not elucidate the relationships among these features.

An important translational application of *XplainScar* is the ability of our model to uncover ECG signatures of LV scar in each HCM patient. To the best of our knowledge, there is no existing method that extracts comprehensive ECG features representing ventricular depolarization and repolarization from all 12 leads of a rest ECG, and provides a signature of scar in the basal, mid and apical LV in HCM. Prior studies have mainly focused on abnormalities in the QRS complex and/or T waves to derive scores that predict LV scar in heart disease^42,49–52^. However, given the heterogeneity in the location and degree of cardiac hypertrophy, fibrosis as well as the diversity of ECG abnormalities in HCM patients, machine learning methods that use all 12 leads of the ECG are best suited for LV scar detection and explanation.

Each lead of the 12-lead ECG records the electrical field generated by myocardial depolarization and repolarization in a specific region of the heart^53^: V1-V2 reflect electrical activity in the septum; V2-V4 reflect anterior wall activity; I, aVL, V5-V6 reflect lateral wall activity; aVR reflects activity in the basal septum and RV outflow tract. Hence, abnormalities in specific leads are useful to localize cardiac pathology such as fibrosis (scar) which can slow impulse propagation and promote dispersion of repolarization^54^. Higher scar burden would be expected to have greater impact on cardiac electrophysiology, which could explain our results of model performance association with LV scar percentage (Fig. 4). An interesting and novel result of our study is the identification of several QRS features in lead aVR for scar prediction in the basal LV (Fig. 5); these results are physiologically accurate but would likely have been missed if manual ECG measurements had been performed, because lead aVR is often ignored by clinicians during ECG analysis^55,56^. Apical LV scar prediction by *XplainScar* was dominated by T wave features in leads V2-V6 (Fig. 5), which has been confirmed by prior ECG-based studies^42,43^. While T wave inversions in several leads are common in HCM, deep T wave inversions are most commonly seen in apical HCM. Our results from *XplainScar* indicate that fibrosis likely plays a role in generation of deep T wave inversion in leads (V4-V6) that reflect electrical activity in the cardiac apex, by causing inversion of the repolarization sequence^54^. Thus, *XplainScar* has the potential to assist clinicians with assessing and monitoring development of electrical and structural LV remodeling in HCM.

### Limitations and future directions

This is a retrospective study, using multi-center ECG data for detection of LV scar at the time of patients’ first clinic visit. While our cohort of 748 patients is substantial for this study, prospective multicenter studies with even larger and more diverse populations are needed to explore the potential of XplainScar for longitudinal scar detection in HCM. While ECG features identified by *XplainScar* vary with LV scar burden, it is not designed to quantify LV scar. Future model development for longitudinal monitoring and quantification of LV scar would be useful to assist with risk stratification for sudden cardiac death, disease management and testing of antifibrotic therapies.

Notably, we excluded patients with late gadolinium enhancement (LGE) confined to right ventricular (RV) insertion points, as this finding may not represent true myocardial fibrosis. However, in a post-hoc analysis of 39 such cases, XplainScar identified 28% as likely having LV scar.  Further research is warranted to clarify these preliminary findings and assess our model’s applicability to HCM patients with LGE located at RV insertion points as well as other locations in the LV..

A key future direction is to enhance XplainScar’s anatomical precision, moving beyond the current basal, mid, and apical segmental analysis, to pinpoint specific myocardial walls (e.g., anterior, lateral). Our dataset, with its granular wall-specific labels, provides the foundation for this advancement. Post-hoc analysis revealed that XplainScar has limited ability to accurately localize scar at the wall level. While septal wall detection showed moderate performance (F1 ~ 68%), anterior wall detection was poor (F1 < 30%), likely due to data imbalance and the model’s design focused on segment-level (basal, mid, apical) detection. A wall-specific prediction model would offer clinicians more detailed insights into disease progressionand, crucially, for guiding therapeutic interventions.

## Conclusions

*XplainScar*, the first explainable ECG-based machine learning method for LV scar detection and localization in HCM, is computationally lightweight and demonstrates high performance with an F1 score > 90%. *XplainScar* identified ECG signatures of scar in the basal, mid, and apical LV, which included a large number of new, physiologically-accurate ECG features.

## Electronic supplementary material

Below is the link to the electronic supplementary material.

Supplementary Material 1

## Data Availability

The datasets analyzed during this study are not publicly available due to privacy and ethical restrictions. However, data may be shared upon reasonable request to Dr. M. Roselle Abraham (roselle.abraham@ucsf.edu), provided that appropriate IRB approvals are obtained.
