# Supplementary material for: Explainable artificial intelligence identifies and localizes left ventricular scar in hypertrophic cardiomyopathy using 12-Lead electrocardiogram
Source: Sci Rep. 2025 Sep 30;15:33918. doi: 10.1038/s41598-025-09282-7 (PMC12484639; doi:10.1038/s41598-025-09282-7)
Supplement: Supplementary file 1 — Supplementary Material 1 [file 41598_2025_9282_MOESM1_ESM.docx]

**Section 1: Data Sources**

All HCM patients met the standard diagnostic criteria for HCM, namely, LVH (maximum wall thickness > 15 mm) in the absence of uncontrolled hypertension, valvular heart disease, and phenocopies such as amyloidosis, storage disorders. All patients underwent deep clinical phenotyping at the time of their first clinic visit to the JH and UCSF HCM-Centers of Excellence (COE). Phenotyping consisted of 12-lead rest and exercise-stress ECGs, rest and exercise-stress echocardiography, magnetic resonance imaging, phenocopy labs, rhythm monitor or device interrogation, detailed history and physical exam; a subset of patients at JH, and most UCSF patients underwent clinical genotyping. Most HCM patients follow up in the HCM-COE at least once per year, and undergo testing (rest/stress echocardiography, rest/stress ECG, rhythm monitor or ICD interrogation) prior to follow up visits.

**1. Electrocardiograms:** Rest ECG data was acquired using General Electric (GE) machines at the JH-HCM-Center and UCSF-HCM-Center. The sampling frequency ranged from 240 to 500 Hz. The ECG data was stored in the MUSE file format after acquisition. This is a standard format within the MUSE cardiology database management system (GE Healthcare). The MUSE file format was converted to comma-separated values (CSV) format to enable efficient data processing using Python packages and machine learning frameworks. Patients in the JH-HCM-Registry with ventricular-paced rhythms and left bundle branch block (LBBB) were excluded ($n=45$).

**2. Magnetic Resonance Imaging (MRI):** Imaging was performed using a 1.5T system (MAGNETOM Avanto; Siemens) at JH, and a 3T system (Signa GE, or Achieva Philips) at UCSF, using an HCM-specific imaging protocol. Scout images in coronal, sagittal, and axial planes were obtained for planning the long- and short-axis views. ECG-gated cine images, including 10-12 short axis views covering the entire left and right ventricles, and single plane 4- and 2-chamber views were acquired. A standard balanced steady state free precession (SSFP) sequence with a temporal resolution of 30-40 msec was used. Late gadolinium enhancement (LGE) images were obtained using a two-dimensional inversion recovery prepared segmented gradient echo sequence, 10 to 15 min after intravenous administration of gadopentate dimeglumine (0.2-mmol/kg). An inversion scout sequence was used to determine the optimal inversion time for nulling normal myocardial signal.

*Magnetic resonance image analysis for LV scar labeling and quantification:* Left ventricular mass and LGE were quantified using QMass software (Version 7.4, Medis medical imaging systems, Leiden, Netherlands). An expert manually traced the endocardial and epicardial borders in each slice of the myocardium (short axis view), dividing it into 16 segments starting from the anterior insertion point of the right ventricle. A region of interest was selected in an area of normal-appearing nulled myocardium, typically the basal lateral wall. Abnormal pixels were identified by considering those with a signal intensity 6 standard deviations (SDs) higher than the mean of normal myocardium [1]. The expert manually edited high signal intensity pixels to exclude artifacts such as blood pool or motion blurring that were erroneously labeled as LGE by the software. The location of LGE (RV insertion points, base, mid, apex) was recorded. LGE was quantified as a percentage of total LV mass. Patients with poor MRI images where LV-LGE could not be quantified reliably, and patients with LGE at RV insertion point(s) were excluded ($n=137$ in JH dataset; $n=39$ in UCSF dataset).

3. **Rest and Stress-Echocardiography**: Rest and stress-echocardiography was performed using GE Vivid 7 ultrasound machines, equipped with a multi-frequency phased-array transducer, using an HCM-specific protocol [2, 3]. A comprehensive analysis of echocardiography data was performed and included LV wall thickness, rest and provoked LV outflow tract gradients (LVOTG), LV systolic and diastolic function [4].

4. **Electronic Health Records (EHR):** Electronic health record data at JH and UCSF, from the initial clinic visit and subsequent follow up visits, spanning an average duration of 2.9 years (range: 1 month to 10 years), was reviewed [5]. In addition to history and physical exam, the treating physician documented information such as medications, arrhythmias, symptoms, co-morbidities, risk factors for sudden cardiac death [6]. The data was reviewed independently for accuracy.

# **Supplemental Table 1.** Patient characteristics in Johns Hopkins and UCSF HCM patient cohorts.

| **Variable** | JH-HCM-COE (n=500) | UCSF-HCM-COE (n=248) |
| --- | --- | --- |
| **Demographics** | | |
| Age (years) | 54.2 ± 15.1 | 57.2 ± 15.3 |
| Male | 343 | 142 |
| Body mass index, kg/m^2^ | 29.4 ± 5.5 | 28.5 ± 5.9 |
| **HCM type** | | |
| Non-obstructive | 146 | 78 |
| Labile-obstructive | 186 | 90 |
| Obstructive | 168 | 80 |
| **NYHA class** | | |
| I | 272 | 78 |
| II-III | 228 | 110 |
| Angina | 217 | 82 |
| Family history of HCM | 93 | 59 |
| History of ICD implantation | 9 | 5 |
| **Risk factors for sudden cardiac death** | | |
| Unexplained syncope | 95 | 36 |
| Family history of sudden cardiac death | 129 | 35 |
| Non-sustained ventricular tachycardia | 52 | 40 |
| ECHO: maximum wall thickness ≥3 cm | 33 | 5 |
| ECHO: left atrial diameter (mm) | 41.6 | 42.4 |
| ECHO: left ventricular ejection fraction (%) | 65.4 ± 8 | 70.1 ± 8 |
| ECHO: Peak rest LVOT gradient (mm Hg) | 30.8 ± 32.8 | 23.8 ± 25.9 |
| ECHO: Peak stress LVOT gradient (mm Hg) | 71.5 ± 52.9 | 51.2 ± 40.2 |
| **Diastolic function** | | |
| ECHO: E/A | 1.3 ± 0.7 | 1.3 ± 0.7 |
| ECHO: E/e´ | 17.9 ± 9.9 | 12.6 ± 6 |
| **Magnetic resonance imaging (MRI)** | | |
| MRI: LV mass index (LV mass / body surface area) | 90 ± 30.7 | 104 ± 34.5 |
| MRI: LV-LGE (% of LV mass) | 9.9 ± 11.7 | 5.7 ± 7.4 |
| **Medications** | | |
| Beta blocker | 375 | 128 |
| Calcium channel blocker | 145 | 54 |
| RAS blockade | 129 | 61 |
| Disopyramide | 14 | 3 |
| Amiodarone | 2 | 8 |
| **Outcomes** | | |
| Follow up (years) | 4.6 | 3.4 |
| New sustained ventricular tachycardia/fibrillation or appropriate ICD discharges | 67 | 42 |
| Atrial fibrillation | 35 | 21 |
| Heart failure | 15 | 18 |
| Death | 12 | 0 |
| **ECGs available for analysis** | 1236 |  |


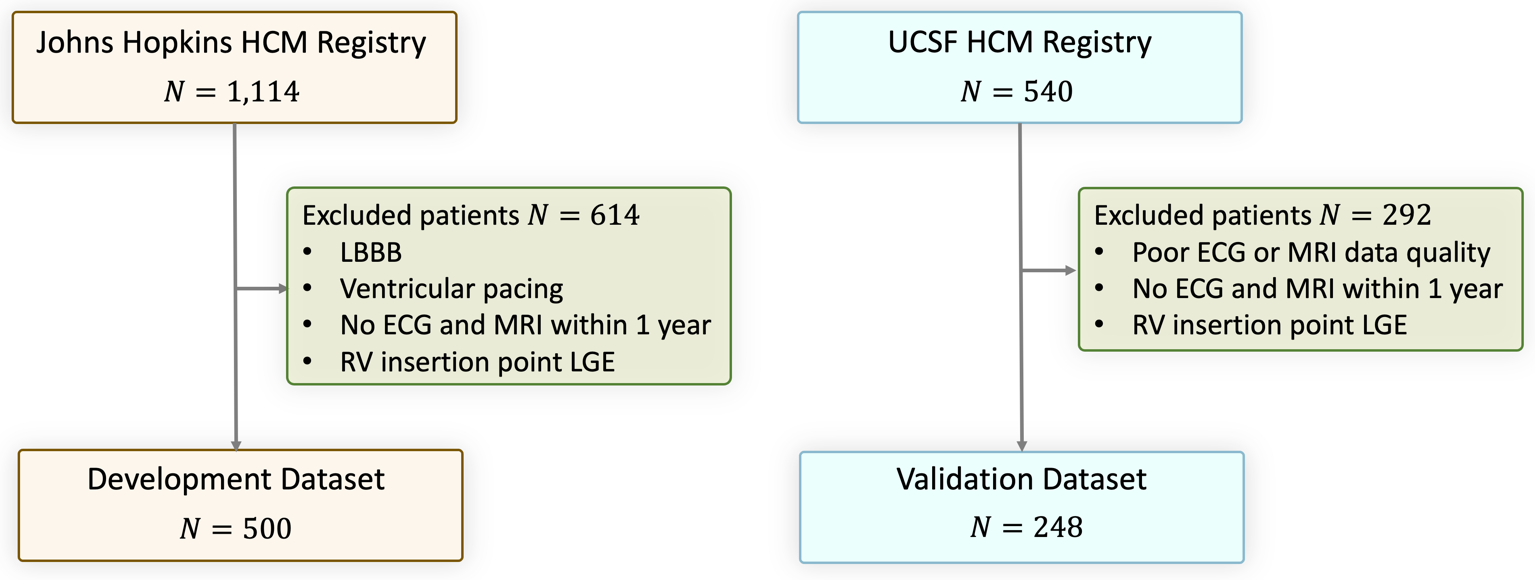


**Supplemental Figure 1.** Patient recruitment and exclusion criteria for our development and validation datasets from Johns Hopkins and UCSF HCM registry.

**Supplemental Table 2** Electronic health record (EHR), echocardiogram (ECHO), and MRI parameters used by supervised neural networks to identify patient's clusters identical to those obtained using unsupervised ECG clustering. Supervised neural networks trained on these parameters can effectively separate HCM patients into groups identical to those identified by using solely patients’ ECG features.

| **EHR Parameters** |
| --- |
| Body Mass Index (kg/m2) |
| History of diabetes |
| History of smoking |
| History of obstructive CAD |
| History of NSVT |
| History of VT/VF |
| History of AFib |
| History of hypertension |
| History of myocardial infarction |
| History of Antitachycardia pacing |
| History of ICD implantation |
| Number of ICD shock in past |
| Outcome: NSVT |
| Outcome: AFib |
| Outcome: Heart failure |
| **ECHO Parameters** |
| Septal maximal wall thickness (mm) |
| LV posterior maximal wall thickness (mm) |
| LV apical wall thickness (mm) |
| LV outflow tract gradient at rest (mmHg) |
| LV outflow tract gradient at peak stress (mmHg) |
| LV diastolic function |
| LV systolic function |
| LV end-systolic volume (ml) |
| LV end-diastolic volume (ml) |
| Mitral valve early-stage flow velocity |
| Mitral valve late-stage flow velocity (m/s) |
| Early mitral inflow deceleration time (ms) |
| LV ejection fraction (%) |
| **MRI Parameters** |
| LGE (% of LV mass) |
| LV mass index |
| LV mass at end-diastole |
| Scar tissue mass (g) |

**Supplemental Table 3.** ECG features extracted by XplainScar from each heartbeat in a 12-lead ECG. Each lead is represented by a 23-dimensional feature vector $V = <\bar{v}_{1}, \bar{v}_{2}, \ldots,\bar{v}_{23}>$ where $\bar{v}_{i}$ is the average of the feature $v_{i}$ in all heartbeats in the lead. By concatenating the feature vectors of the individual leads, a 12-lead ECG is represented by a $12 \times23=276$-dimension vector.

| Feature | Unit | Description |
| --- | --- | --- |
| QRS complex features | | |
| Q, R, and S amplitudes | $\mathrm{mV}$ | Amplitude of the lowest point of Q and S waves and the peak of the R wave. |
| Duration | $\mathrm{second}$ | Duration of the segment between the Q-onset and S-offset. The R-wave’s boundary is replaced if any of Q or S wave is absent |
| Non-terminal duration | $\mathrm{second}$ | Duration of the non-terminal portion of QRS defined as the interval between the QRS onset and minimum point of the S-wave (or R-peak, if S-wave is absent) |
| Terminal duration | $\mathrm{second}$ | Duration of the terminal portion of QRS defined as the interval between the minimum point of S-wave (or R-peak, if S-wave is absent) and QRS offset. |
| Q slope | $\mathrm{mV}/\mathrm{second}$ | Slope of the line intercepting QRS onset and minimum point of Q-wave. If the Q-wave is absent, the slope is set to zero. |
| R upstroke slope | $\mathrm{mV}/\mathrm{second}$ | Slope of the line intercepting the minimum point of Q-wave and R-peak. If Q-wave is absent, the slope is calculated from QRS-onset. If the R-wave is absent, the slope is set to zero. |
| RS slope | $\mathrm{mV}/\mathrm{second}$ | Slope of the line intercepting R-peak and minimum point of S-wave |
| Number of notches | - | Number of notches in the QRS complex |
| Number of terminal notches | - | Number of notches in the terminal portion of the QRS complex |
| Maximum notch prominence | $\mathrm{mV}$ | Maximum notch prominence defined as the vertical distance between the notch and its lowest contour line |
| Energy | $\mathrm{mV}^{2}\times second$ | Energy of QRS complex defined as $\int_{t=\mathrm{QRS}_{\mathrm{on}}}^{t=\mathrm{QRS}_{\mathrm{off}}} {\vert x(t)\vert}^{2}\mathrm{dt}$ where $x(t)$ is the amplitude of ECG at $t$. |
| Area Under Curve | $mV\times second$ | Area under the QRS complex defined as $\int_{t=\mathrm{QRS}_{\mathrm{on}}}^{t=\mathrm{QRS}_{\mathrm{off}}} x(t)dt$ where $x(t)$ is the amplitude of ECG at $t$. |
| ST-segment features | | |
| Slope | $\mathrm{mV}/\mathrm{second}$ | Slope of the line fitted to the segment between the end of QRS and the start of T-wave. |
| Onset and offset elevation | $\mathrm{mV}$ | Elevation of the onset and offset of the ST segment based on the baseline amplitude |
| T-wave features | | |
| Peak amplitude | $\mathrm{mV}$ | Amplitude of the extremum point of the T-wave. |
| Duration | $\mathrm{second}$ | Duration of T-wave. |
| Terminal duration | $\mathrm{second}$ | Duration of the interval between the extremum point of T-wave and T-offset. |
| Energy | $\mathrm{mV}^{2}\times second$ | Energy of T-wave defined as $\int_{t=T_{\mathrm{on}}}^{t=T_{\mathrm{off}}} {\vert x(t)\vert}^{2}\mathrm{dt}$ where $x(t)$ is the amplitude of ECG at $t$. |
| Area Under Curve | $mV\times second$ | Area under the T-wave defined as $\int_{t=T_{\mathrm{on}}}^{t=T_{\mathrm{off}}} x(t)dt$ where $x(t)$ is the amplitude of ECG at $t$. |
| TP-segment features | | |
| TP slope | $\mathrm{mV}/\mathrm{second}$ | Slope of the line fitted on the segment between the end of T-wave and start of P-wave. |

## **Unsupervised ECG Clustering**


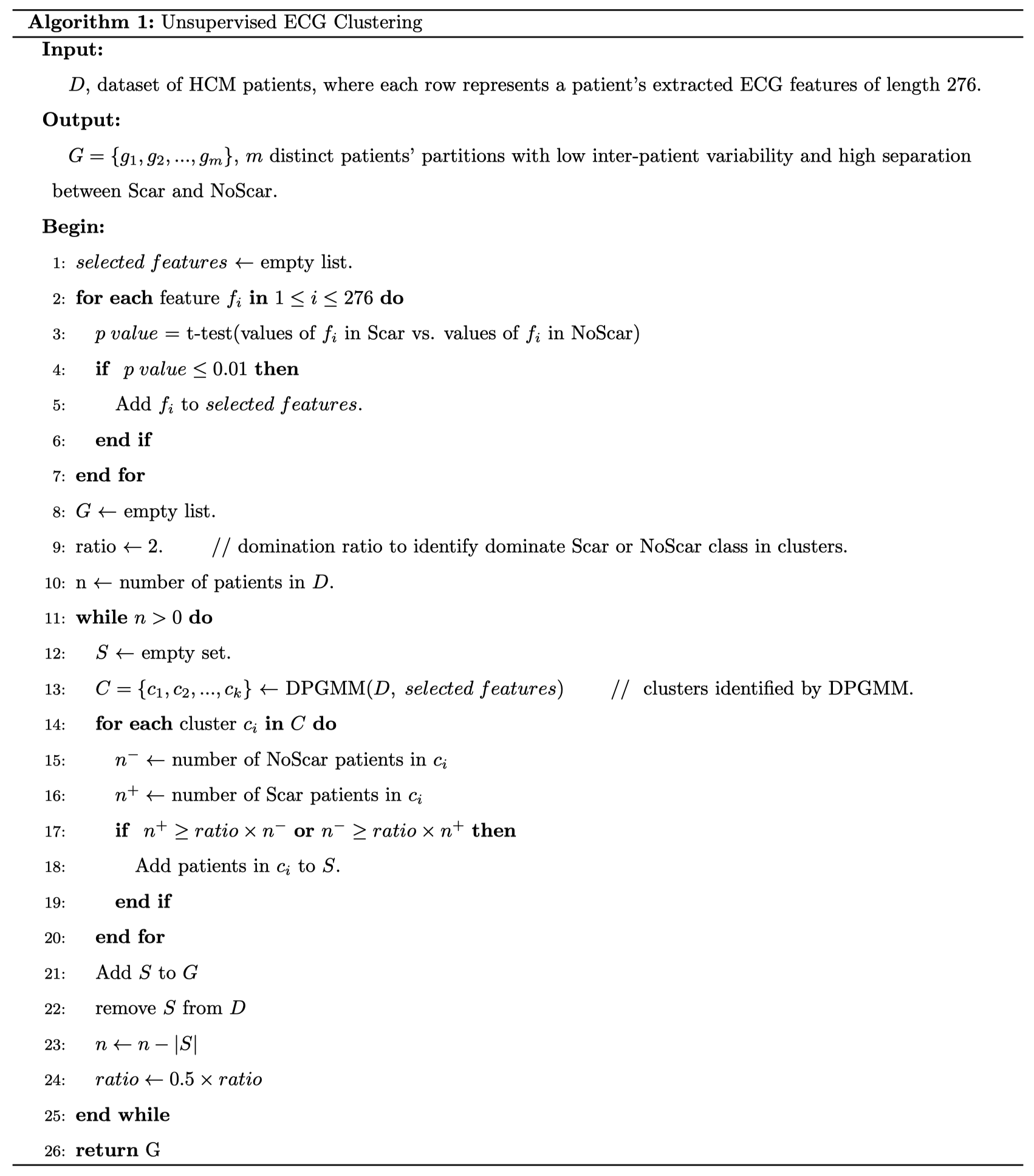


**Details on Explaining Model Predictions using Shapley Additive Explanation**

Given our entire trained machine learning model $f$ and an input ECG $x\in\mathbb{R}^{M}$ with $M$ extracted ECG features, we seek to explain the prediction $f(x)$ by assigning importance score to each feature in $x$. We use the Shapley value approach, a theoretically justified solution from cooperative game theory, to compute the scores. It is the only allocation of scores among various features in $x$ that can be considered *fair* and *explainable* as it obeys the following properties:

1. The Shapley value of a feature whose value does not impact the prediction is zero.
2. Shapley values of any two features with symmetrical impact are equal.
3. Let $h\left( x \right)=f\left( x \right)+g(x)$. The Shapley value of a feature in $h(x)$ prediction equals the sum of the Shapley values in $f(x)$ and $g(x)$ predictions. We used this property to compute the Shapley value of ECG features of scar in the entire LV by summing Shapley values obtained from basal, mid, and apical LV-scar detection tasks.
4. Shapley values are implicitly normalized, making them easier to compare across different predictions.

The Shapley value $\phi_{i}(f, x)$ is a single numerical value showing the contribution of feature $x_{i}$ in the prediction $f(x)$ and is computed as follows:

$\phi_{i}(f, x)=\frac{1}{n}\sum_{S\subseteq M\setminus\{i\}} \left( \begin{aligned} n-1 \\ |S| \end{aligned} \right)^{-1}(f_{x}(S\cup\{i\})-f_{x}(S))$,

where $S$ is a subset of features excluding the feature $i$, and $f_{x}(S)$ is the model’s prediction using only features in $S$. This solution requires retraining the model $f$ on all feature subsets, resulting in $2^{M}$ different models (in game theory, this is equivalent to $2^{M}$ different coalitions that $M$ players can form to win a prize). As the classic approach is practically not feasible in machine learning, we use well-known Shapley Additive Explanation (SHAP) [7], where Shapley values are estimated using a weighted linear regression as follows:

1. Generate a dataset $D_{z}$ consisting of $K$ binary vectors $z\in{\{0,1\}}^{M}$.
2. Map each vector $z$ to the ECG feature space by replacing 1’s in $z$ with their corresponding feature values in $x$ and replacing 0’s with feature values of another ECG sampled randomly from the training set. Let $h_{x}(z)$ denote this mapping function.
3. Compute the weight for the vector $z$ as follows:

$$\pi\left( z \right)=\frac{(M-1)}{\left( M\text{ choose }\left| z \right| \right)\left| z \right|\left( M-\left| z \right| \right)}$$

where $\left| z \right|$ is the number of non-zero elements in $z$.

1. Fit a linear model $g(z)=\phi_{0}+\sum_{i=1}^{M} \phi_{i}z_{i}$ on $D_{z}$ such that the following loss function is minimized:

$$L\left( f,g,\pi\right)=\sum_{z\in D_{z}} \left[ f\left( h_{x}\left( z \right) \right)-g\left( z \right) \right]^{2}\pi\left( z \right)$$

1. Return coefficients of $g$ as Shapley values.

The function $g$ is commonly referred to as ‘*explanation* model’, allocating Shapley values to each individual ECG feature in $x$ and showing their contribution to the prediction $f(x)$. Each vector $z$ can be viewed as a coalition of ECG features where 0’s show that the corresponding feature is not present in the coalition. The coalition space $D_{z}$ has the maximum size of $K=2^{M}$, but each coalition $z$ can be mapped to several ECG feature vectors in step 2 by replacing 0’s in $z$ with different random draws from the training set. The weight $\pi\left( z \right)$ is larger for $z$’s with few 1’s (small coalitions) and those with many 1’s (large coalitions). This enforces the explanation model $g$ to learn more from the effect of ECG features when they are in isolation.

The above-mentioned approach is implemented by Kernel Explainer in the SHAP framework. The authors of SHAP proved that the coefficients of $g$ recover the Shapley values properties; as such, we employ it for explaining our LV scar detection framework.

[1] P. E. Bravo *et al.*, "Late gadolinium enhancement confined to the right ventricular insertion points in hypertrophic cardiomyopathy: an intermediate stage phenotype?," *European Heart Journal-Cardiovascular Imaging,* vol. 17, no. 3, pp. 293-300, 2016.

[2] I. Pozios *et al.*, "Rest and stress longitudinal systolic left ventricular mechanics in hypertrophic cardiomyopathy: Implications for prognostication," *Journal of the American Society of Echocardiography,* vol. 31, no. 5, pp. 578-586, 2018.

[3] H. C. Luo *et al.*, "Exercise heart rates in patients with hypertrophic cardiomyopathy," *The American journal of cardiology,* vol. 115, no. 8, pp. 1144-1150, 2015.

[4] D. Y. Lu *et al.*, "Clinical outcomes in patients with nonobstructive, labile, and obstructive hypertrophic cardiomyopathy," *Journal of the American Heart Association,* vol. 7, no. 5, p. e006657, 2018.

[5] M. Bhattacharya *et al.*, "Identifying ventricular arrhythmias and their predictors by applying machine learning methods to electronic health records in patients with hypertrophic cardiomyopathy (HCM-VAr-risk model)," *The American journal of cardiology,* vol. 123, no. 10, pp. 1681-1689, 2019.

[6] D. Y. Lu *et al.*, "Sex-specific cardiac phenotype and clinical outcomes in patients with hypertrophic cardiomyopathy," *American heart journal,* vol. 219, pp. 58-69, 2020.

[7] S. M. Lundberg and S.-I. Lee, "A unified approach to interpreting model predictions," *Advances in neural information processing systems,* vol. 30, 2017.
